# Prevalence and Causes of Stigmatization Among Patients With Chronic Skin Diseases in Saudi Arabia

**DOI:** 10.7759/cureus.59373

**Published:** 2024-04-30

**Authors:** Yassmeen Hmoud Alblowi, Ahmed A Alsaati, Amirah Saleh Alzubaidi, Sahar Saud Alsifri, Yousef AlHarthi, Moteb Khalaf Alotaibi

**Affiliations:** 1 Department of Emergency Medicine, King Khaled Hospital, Ministry of Health, Tabuk, SAU; 2 Department of Dermatology, College of Medicine, King Faisal University, Al-Hofuf, SAU; 3 Department of Medicine and Surgery, College of Medicine, Umm Al-Qura University, Al-Qunfudhah, SAU; 4 Department of Dermatology, Faculty of Medicine, King Abdulaziz University, Jeddah, SAU; 5 Department of Dermatology and Dermatologic Surgery, Prince Sultan Military Medical City, Ministry of Defence, Riyadh, SAU; 6 Department of Medicine, Unaizah College of Medicine and Medical Sciences, Qassim University, Unaizah, SAU

**Keywords:** vitiligo, stigmatization level, skin diseases, psoriasis, dermatology

## Abstract

Background: Skin diseases can lead to stigmatization with negative consequences for patients' quality of life and mental health.

Aim: The aim of this study was to estimate the prevalence of stigmatization experienced by patients with vitiligo, psoriasis, acne, rosacea, or atopic dermatitis and to assess the relationships between the level of stigmatization and patient characteristics.

Methods: This cross-sectional study included adult patients with vitiligo, psoriasis, acne, rosacea, or atopic dermatitis attending the dermatology clinics of various general hospitals in Saudi Arabia. Stigma levels were assessed using the six-item Stigma Scale.

Results: The prevalence of stigmatization was 90.4% among the 280 patients included. Multiple regression analyses revealed the factors that independently and significantly increased the level of stigmatization. These included male gender (B = 4.300, 95%CI 3.407-5.192, P <0.001), positive family history of skin conditions (B = 2.267, 95%CI 1.139-3.395, P <0.001), number of skin diseases (B = 2.357, 95%CI 0.998-3.716, P = 0.001), and presence of facial lesions (B = 2.455, 95%CI 1.206-3.705, P<0.001).

Conclusions: The prevalence of stigmatization is high among patients with chronic skin diseases in Saudi Arabia. Identifying patients at risk for high levels of stigmatization may allow them to be provided with appropriate social and psychological support.

## Introduction

Stigmatization is a phenomenon that has existed for centuries and can be related to any form of “otherness” [1]. It refers to a community's negative perception of an individual or group of individuals based on their social, physical, behavioral, or mental characteristics [2]. Stigmatization of individuals includes labeling them as different, negative stereotyping, categorization and segregation, and discrimination against them. All of these reactions lead to social isolation [3]. Stigmatization has been related to a variety of health conditions and diseases, including mental illness, obesity, skin diseases, and acquired immune deficiency [4-6].

Skin diseases such as vitiligo, psoriasis, acne, rosacea, and atopic dermatitis affect a considerable proportion of the Saudi population. Previous studies reported that the prevalence rates of vitiligo and psoriasis were 6% [7] and 5.33% [8], respectively, in the Saudi population.

Skin diseases, especially those that cause visible skin lesions, can significantly affect the physical appearance of patients. This in turn influences community attitudes toward these patients, leading to discrimination, negative emotions, and social rejection [2]. In addition, these patients may suffer from low self-esteem and feelings of inadequacy, increasing the risk of psychosocial comorbidities such as depression, anxiety, and suicidal ideation [9]. Patients with chronic dermatological conditions are prone to feelings of stigma and impaired health-related quality of life [10-12].

The causes of stigmatization of patients with chronic skin diseases usually stem from misconceptions, such as the belief that the disease is caused by inadequate personal hygiene. Such misconceptions may lead to blaming the affected individual for his or her condition. Other misconceptions may include the belief that the family has bad genes, which can lead to avoidance and social isolation of all members of that family [10,13,14].

Although skin diseases have a significant negative impact on patients' lives, these consequences are often overlooked and under-treated [15,16]. This can lead to poor patient compliance, inadequate therapeutic response, and unfavorable disease outcomes [17].

There is limited data on the experience of stigma among patients with chronic visible skin diseases in Saudi Arabia. Assessing the level of stigma experienced by patients with dermatoses is crucial in providing them with appropriate health services. The aim of this study was to estimate the prevalence of stigmatization in Saudi Arabia experienced by patients with vitiligo, psoriasis, acne, rosacea, or atopic dermatitis and to assess the relationship between the level of stigmatization and various demographic and clinical characteristics of the patients.

## Materials and methods

This was a cross-sectional survey study conducted among Saudi patients with chronic skin diseases who were attending dermatology clinics at different general hospitals in Saudi Arabia. Data collection took place between March and October 2023. The study was approved by the Ethics Committee of the Deanship of Scientific Research, Qassim University, Qassim, Saudi Arabia (approval number: 035-1449). Participants were informed about the study's objectives, methodology, risks, and benefits, and were asked to provide informed consent before completing the questionnaire. Anonymous data collection was utilized to ensure the confidentiality of the participants.

Inclusion ad exclusion criteria

The study enrolled male and female Saudi patients, 18 years of age and older, who had one of the following chronic skin diseases: vitiligo, psoriasis, acne, rosacea, or atopic dermatitis. Non-Saudi subjects, patients under 18 years of age, and those with skin diseases not listed in the inclusion criteria were excluded from the study.

Sample size calculation

The sample size was calculated using G*power software version 3.1.9.2 for Windows (Heinrich-Heine-Universität Düsseldorf, Düsseldorf, Germany). The calculation was based on the need to perform multiple regression, assuming an alpha error level of 0.01, a power of 0.95, a maximum number of predictors tested of 13, and an effect size of 0.15 (representing a medium effect size of the change in R^2^). The calculated sample size was 240. To adjust for incomplete responses, 40 participants were added, resulting in a final sample size of 280.

Sampling technique

The participants were selected through a simple random sampling technique by generating random numbers using an online random number generator (https://randomnumbergenerator.org/). In each clinic, a selected random number was matched with the last four digits of the medical records.

Data collection

A structured, self-administered questionnaire (See Appendices) was distributed directly to the patients attending the dermatology clinics. The questionnaire consisted of three parts. The first part collected the participants’ sociodemographics including gender, age group, marital status, education level, employment, healthcare employment and/or education, monthly income, and nationality. The second part focused on the participants’ clinical characteristics, including the diagnosis, the age of onset, the disease duration, and having facial lesions or a family history. The third part assessed the participants’ stigmatization using the six-item Stigmatization Scale [18]. The scale used in this study was the Arabic version prepared and validated by Dimitrov et al. [19]. Each question was rated on a four-point Likert scale (1, not at all; 2, sometimes; 3, very often; 4, always). The minimum and maximum overall stigmatization scores are 6 and 24, respectively. Higher scores indicate a greater feeling of stigmatization [20].

Statistical analysis

Data analysis was carried out using IBM SPSS Statistics for Windows, Version 22.0 (Released 2013; IBM Corp., Armonk, New York, United States). Categorical data (e.g., gender, age groups, and marital status) were presented as frequencies and percentages, while numerical data (e.g., the score for stigmatization) were presented as means and standard deviations (SDs). Comparisons of the scores were carried out using the independent samples T-test (for two groups) or one-way analysis of variance (for more than two groups) if the data were normally distributed. For non-normally distributed data, the Mann-Whitney test was used to compare two groups. Multiple regression was performed for the relevant variables that had a p-value <0.1 in the univariate analysis. A p-value <0.05 indicated statistical significance.

## Results

Out of the 788 individuals invited to participate in the study, only 781 responded. Of those, 399 participants were excluded due to having no history of chronic skin disease. The remaining 382 participants reported a diagnosis of a skin disease, but 85 did not complete the questionnaire and 17 were non-Saudi, resulting in a final sample of 280 participants. All were Saudi patients.

The majority of patients were between the ages of 21 and 64. Gender distribution was almost equal. The majority of participants were single (68.9%), while 28.6% were married. Respondents had a high level of education, with 70.0% having a university degree and 9.6% having a postgraduate degree. The survey results indicate that 47.5% of the respondents were students, while 41.8% were employed. More than half of the respondents (51.8%) had employment and/or education in healthcare. In terms of monthly income, 51.1% of the respondents reported earning below 5,000 Saudi Riyals (SAR). The most commonly reported skin disorder was acne (65.7%), followed by atopic dermatitis (22.5%), psoriasis (15.4%), vitiligo (10.4%), and rosacea (5.4%). Additionally, 8% of the respondents reported having two chronic dermatological conditions, and 5.4% reported having three conditions. In this study, it was found that 57.9% of patients developed the skin disease at the age of 20 or younger, while 39.6% developed it between the ages of 21 and 64. Only 2.5% of patients developed the disease after the age of 64. The majority of participants (71.4%) received treatment for the skin disease. More than half of the participants (54.6%) had a positive family history of skin disease, with acne being the most commonly reported familial condition (35.7%), followed by psoriasis (18.6%), and atopic dermatitis (15.4%). Additionally, approximately one-third of participants had lesions on their face (Table 1).

**Table 1:** Sociodemographic Characteristics of the Participants (N = 280)

Sociodemographic characteristics		n (%)
Age, year	≤ 20	43 (15.4%)
	21-64	235 (83.9%)
	≥ 65	2 (0.7%)
Gender	Female	158 (56.4%)
	Male	122 (43.6%)
Marital status	Single	193 (68.9%)
	Married	80 (28.6%)
	Widow/divorced	7 (2.5%)
Education level	Primary/intermediate school	6 (2.1%)
	Secondary school	51 (18.2%)
	University degree	196 (70.0%)
	Postgraduate studies	27 (9.6%)
Employment	Unemployed	20 (7.1%)
	Student	133 (47.5%)
	Employed	117 (41.8%)
	Retired	10 (3.6%)
Employment and/or education in healthcare	No	135 (48.2%)
	Yes	145 (51.8%)
Monthly income, Saudi Riyal	< 5,000	143 (51.1%)
	5,000 - 10,000	64 (22.9%)
	> 10,000	73 (26.1%)
Diagnosis	Vitiligo	29 (10.4%)
	Psoriasis	43 (15.4%)
	Acne	184 (65.7%)
	Atopic dermatitis	63 (22.5%)
	Rosacea	15 (5.4%)
Number of skin disorders	1	242 (86.4%)
	2	23 (8.2%)
	3	15 (5.4%)
Age at onset, years	≤20	162 (57.9%)
	21-64	111 (39.6%)
	≥65	7 (2.5%)
Receiving treatment for skin disease	No	80 (28.6%)
	Yes	200 (71.4%)
Duration of the disease	<3 months	70 (25.0%)
	≥3 months	210 (75.0%)
Family history of a chronic skin disease	No	127 (45.4%)
	Yes	153 (54.6%)
	Vitiligo	26 (9.3%)
	Psoriasis	52 (18.6%)
	Acne	100 (35.7%)
	Atopic dermatitis	43 (15.4%)
	Rosacea	12 (4.3%)
Lesions in the face	No	190 (67.9%)
	Yes	90 (32.1%)

**Figure 1:**
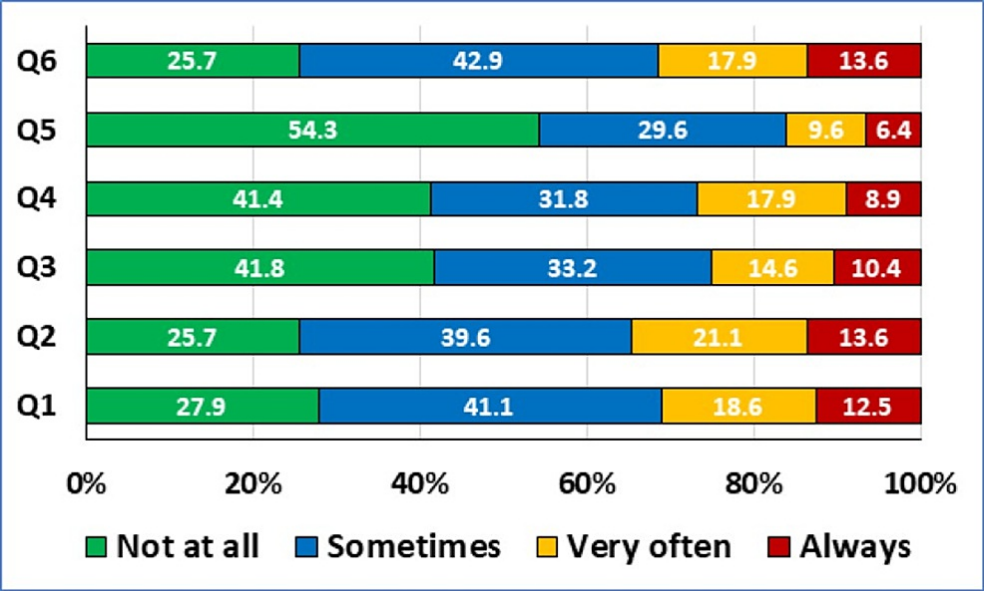
Responses to the Stigmatization Score Questions (N=280) Q1: Others are not attracted to me due to my skin disease; Q2: I think that others stare at my skin disease; Q3: Others feel uncomfortable touching me due to my skin disease; Q4: Other people think that my skin disease is contagious; Q5: Other people avoid me due to my skin disease; Q6: Other people sometimes make annoying comments about my skin disease Data presented as percentages

The participants' responses to the stigmatization questionnaire are displayed in Figure 1.

**Figure 2:**
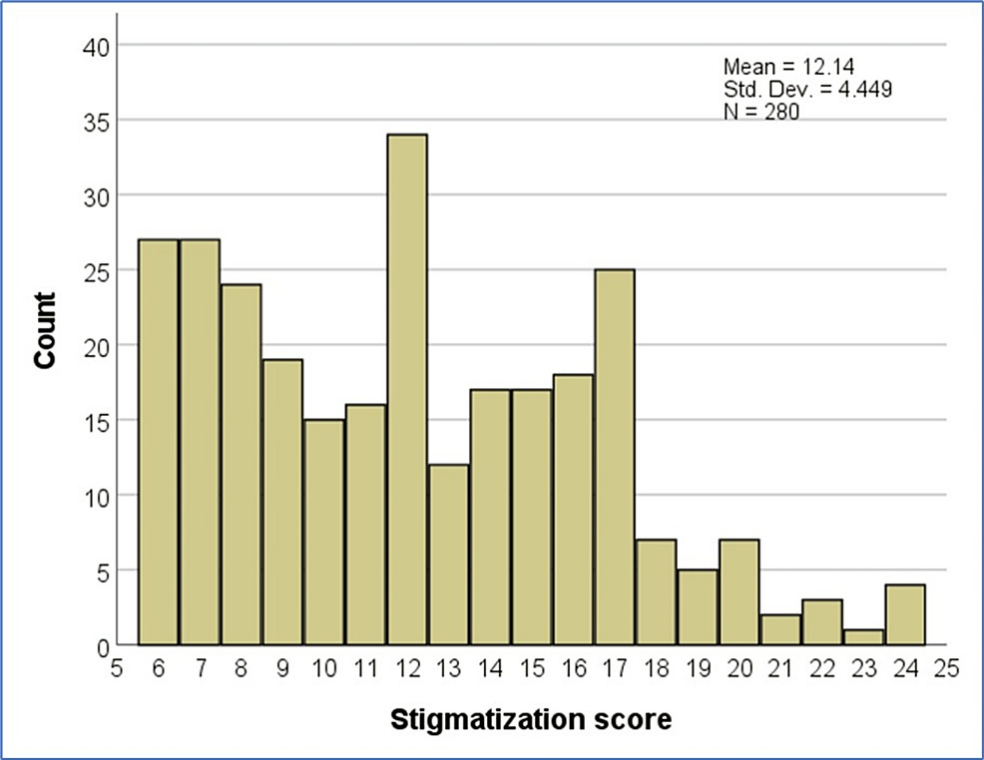
Histogram Showing the Frequency of Participants for Each Value of the Stigmatization Score Std. Dev: standard deviation (SD)

The calculated stigmatization score had a mean of 12.14 and a standard deviation of 4.45 (Range, 6-24). Twenty-seven respondents (9.6%) had the lowest scores (each given a score of 6) because they answered negatively to all six questions of the stigma score and were considered to have no stigma. Meanwhile, the majority of respondents had high scores because they responded positively to one or more of the questions and were considered to be stigmatized (they received scores ranging from 7 to 24). The prevalence of stigmatization due to the chronic skin condition was 90.4% (Figure 2).

Male participants had a significantly higher mean score than females (12.77 ± 4.46 vs. 11.65 ± 4.39, p = 0.036). Additionally, patients with a positive family history had significantly higher scores compared to those with a negative history (12.75 ± 4.53 vs. 11.40 ± 4.25, p = 0.012). Patients with a family history of vitiligo, psoriasis, and acne had higher scores (14.38 ± 4.65 vs. 11.91 ± 4.37, p = 0.007; 13.15 ± 4.11 vs. 11.90 ± 4.50, p = 0.036; 13.13 ± 4. 51 vs. 11.58 ± 4.33, p = 0.005, respectively), while those with a family history of atopic dermatitis had significantly lower scores (10.02 ± 4.17 vs. 12.52 ± 4.40, p<0.001). There were no significant differences found among the respondents in terms of age, marital status, educational level, employment status, healthcare employment or education, monthly income, or family history of rosacea (all p-values >0.05) (Table 2).

**Table 2:** Comparison of the Stigmatization Score in Relation to the Participants’ Sociodemographic Data F: One-way analysis of variance (ANOVA); SD: standard deviation; t: Independent samples T-test; Z: Mann-Whitney test *significant at p<0.05

Sociodemographic factors		Mean ± SD	Test statistic	p-value
Age, year	≤ 20	12.28 ± 4.54	1.110 F	0.331
	21 – 64	12.15 ± 4.44		
	≥ 65	7.50 ± 2.12		
Gender	Female	11.65 ± 4.39	2.111 t	0.036*
	Male	12.77 ± 4.46		
Marital status	Single	11.91 ± 4.60	1.178 F	0.309
	Married	12.53 ± 3.92		
	Widow/divorced	14.00 ± 5.86		
Education level	Primary/intermediate school	12.67 ± 5.32	0.906 F	0.439
	Secondary school	11.82 ± 4.26		
	University degree	12.36 ± 4.52		
	Postgraduate studies	10.96 ± 4.08		
Employment	Unemployed	12.35 ± 4.85	0.652 F	0.587
	Student	12.24 ± 4.90		
	Employed	11.86 ± 3.88		
	Retired	13.50 ± 3.69		
Employment and/or education in healthcare	No	12.10 ± 4.33	0.143 t	0.887
	Yes	12.17 ± 4.57		
Monthly income, Saudi Riyal	< 5,000	12.22 ± 4.84	2.499 F	0.086
	5,000 - 10,000	12.84 ± 4.31		
	> 10,000	11.36 ± 3.62		
Family history of a chronic skin disease	No	11.40 ± 4.25	2.540 t	0.012*
	Yes	12.75 ± 4.53		
Family history: Vitiligo	No	11.91 ± 4.37	2.737 t	0.007*
	Yes	14.38 ± 4.65		
Family history: Psoriasis	No	11.90 ± 4.50	2.095 Z	0.036*
	Yes	13.15 ± 4.11		
Family history: Acne	No	11.58 ± 4.33	2.822 t	0.005*
	Yes	13.13 ± 4.51		
Family history: Atopic dermatitis	No	12.52 ± 4.40	3.450 t	<0.001*
	Yes	10.02 ± 4.17		
Family history: Rosacea	No	12.16 ± 4.42	0.505 t	0.614
	Yes	11.50 ± 5.28		

We compared the stigmatization scores of respondents with and without vitiligo, psoriasis, and atopic dermatitis. We found significantly higher scores in patients diagnosed with vitiligo (p = 0.024) and psoriasis (p = 0.011) compared to those without these diseases. Conversely, the mean score was significantly lower in patients diagnosed with atopic dermatitis (p<0.001). Patients with facial skin lesions had a significantly higher mean score than those without (13.64 ± 4.74 vs. 11.42 ± 4.13, p<0.001). There were no significant differences regarding the diagnosis of acne or rosacea, the number of skin diseases, the age at onset of the disease, receiving treatment, or disease duration (all p-values >0.05) (Table 3).

**Table 3:** Comparison of the Stigmatization Score in Relation to the Dermatological Disease-Related Data F: One-way analysis of variance (ANOVA); SD: standard deviation; t: Independent samples T-test *significant at p<0.05

Disease-related data		Mean ± SD	Test statistic	p-value
Diagnosis				
Vitiligo	No	11.93 ± 4.46	2.268 t	0.024*
	Yes	13.90 ± 3.96		
Psoriasis	No	11.85 ± 4.49	2.565 t	0.011*
	Yes	13.72 ± 3.91		
Acne	No	11.81 ± 4.53	0.878 t	0.381
	Yes	12.30 ± 4.41		
Atopic dermatitis	No	12.62 ± 4.39	3.461 t	<0.001*
	Yes	10.46 ± 4.28		
Rosacea	No	12.24 ± 4.44	1.617 t	0.107
	Yes	10.33 ± 4.39		
Number of skin disorders	1	12.15 ± 4.49	2.478 F	0.086
	2	10.74 ± 4.09		
	3	14.00 ± 3.74		
Age at onset, year	≤ 20	12.26 ± 4.82	2.061 F	0.129
	21 - 64	11.77 ± 3.88*		
	≥ 65	15.14 ± 2.54*		
Receiving treatment for skin disease	No	11.57 ± 4.17	1.336 t	0.183
	Yes	12.36 ± 4.55		
Duration of the disease, month	< 3	12.31 ± 4.81	0.387 t	0.699
	≥ 3	12.08 ± 4.33		
Lesions in the face	No	11.42 ± 4.13	4.009 t	<0.001*
	Yes	13.64 ± 4.74		

Multiple regression analysis was conducted to evaluate the factors that independently and significantly impact the stigmatization score. Only variables with a p-value less than 0.1 in Tables 2 and 3 were included in the model, excluding family history of specific diseases and actual diagnoses, as more than 13% of participants had multiple conditions. The stigmatization score was significantly increased by independent factors such as male gender (B = 4.300, 95%CI 3.407-5.192, P <0.001), positive family history of skin conditions (B = 2. 267, 95%CI 1.139-3.395, P <0.001), the number of skin diseases (B = 2.357, 95%CI 0.998-3.716, P = 0.001), and the presence of facial lesions (B = 2.455, 95%CI 1.206-3.705, P<0.001) (Table 4).

**Table 4:** Multiple Regression of Factors Affecting the Stigmatization Score B: unstandardized regression coefficient; CI: confidence interval; t: T-test * significant at p<0.05

Independent variables	B	95% CI	t	p-value
Gender Male vs. Female	4.300	3.407 to 5.192	9.482	<0.001*
Monthly income, Saudi Riyal	0.545	-0.061 to 1.152	1.770	0.078
Family history of a chronic skin disease	2.267	1.139 to 3.395	3.955	<0.001*
Number of skin disorders	2.357	0.998 to 3.716	3.414	0.001*
Lesions in the face	2.455	1.206 to 3.705	3.869	<0.001*

## Discussion

In dermatological conditions with visible skin lesions, it is important to understand the extent and predictors of perceived stigma to guide the provision of necessary medical and social care. While the prevalence and impact of perceived stigma in dermatological diseases are high, there is limited knowledge about perceived stigma in skin conditions other than psoriasis [6]. A recent review of stigma in visible skin conditions revealed that 61% of the studies analyzed focused on patients with psoriasis. This emphasizes the necessity for further research into other skin conditions to identify common and specific factors [6].

The study included 280 participants with chronic skin diseases. The most commonly reported skin disorders were acne (65.7%), atopic dermatitis (22.5%), psoriasis (15.4%), vitiligo (10.4%), and rosacea (5.4%). Approximately 13% of the respondents reported having more than one chronic skin condition.

In the present study, 90.4% of the participants reported experiencing stigmatization due to their skin disease. This rate is consistent with the findings of Hrehorów et al., who reported that only 9.8% of patients with psoriasis did not experience stigma [1]. However, a more recent study by van Beugen et al. reported a lower prevalence of stigmatization at 73% [20].

Regarding the relationship between the severity of stigmatization and patients' sociodemographic factors, the results of both univariate and multivariate analyses in this study showed significantly higher stigmatization scores in participants who were male and had a positive family history. The relationship between gender and stigmatization is controversial in the literature. Some studies report a significant association with female patients [9,11], while others report higher scores in males [21-23]. However, several studies have reported a lack of significant association [9,20,24,25].

In our study, the severity of stigmatization was not significantly affected by the respondent's age, marital status, educational level, employment, or monthly income. However, other studies have found that patients with visible skin diseases had higher stigma scores associated with lower age and unemployment [6,20]. Additionally, one study reported a significant correlation between being single and perceived stigmatization [23].

The current study revealed that patients diagnosed with vitiligo or psoriasis had significantly higher stigmatization scores, whereas patients with atopic dermatitis had significantly lower scores in the univariate analysis. In the multiple regression analysis, only a diagnosis of psoriasis was found to independently and significantly increase the stigmatization score. No significant differences were observed in patients diagnosed with acne or rosacea. The literature indicates that stigmatization levels vary across different skin diagnoses, with psoriasis being associated with higher levels of stigma than other diseases. Previous studies have reported that patients with psoriasis experience higher levels of perceived stigmatization compared to patients with atopic dermatitis. The studies reported higher discrimination in patients with psoriasis compared to those with vitiligo. Additionally, they found a stronger correlation of stigma with acne than with atopic dermatitis [26-28].

The mean stigmatization score was higher in patients with three skin diseases, but the result in the univariate analysis was slightly above the significance level of 0.05. When evaluated in the multivariate model, an increased number of skin diseases was significantly correlated with an increased stigmatization score.

We found no significant difference in the stigmatization scores with respect to age at onset of the disease, receiving treatment, or disease duration. In contrast, Germain et al. found that a younger age at diagnosis was associated with higher scores of stigmatization [6]. Previous studies have also reported a significant correlation between higher levels of stigma and/or impact and longer disease duration [9,20,23,29].

In the current study, patients with facial skin lesions had a significantly higher stigmatization score than those without, as shown in both univariate and multivariate analyses. This finding is consistent with previous research [6] and is attributed to the visibility of the lesions, which plays a key role in inducing stigma. In addition, facial lesions may cause disfigurement, which further increases perceived stigma.

Discrepancies in the results of studies assessing stigmatization in skin diseases may stem from variations in the diagnoses of the skin disorders, their severity and visibility, and sociocultural differences between communities. One study found that stigma-precipitating factors differed between patients with vitiligo and psoriasis with dark and light skin, with skin shade having a significant impact [30].

The present study estimated the prevalence of stigmatization in patients with chronic skin diseases and identified the factors that significantly affected the level of stigma. The generalizability of our findings may be limited by the standards and cultural properties of Saudi society. In addition, the questionnaire did not include participants with comorbidities, grading of disease severity, or information about the type and color shade of participants, which could potentially affect the level of stigmatization. Moreover, owing to the study design and tools, we had no information on the extent of lesions on different body parts other than the face, which could impact the level of stigmatization. Furthermore, the study used a cross-sectional design, which limits the ability to collect data on changes in stigmatization levels within patients over time and with disease progression.

## Conclusions

Identifying patients at high risk of experiencing stigma may help to provide them with appropriate social, psychological, and healthcare support. Future studies should preferably be longitudinal to assess changes in stigma levels over time. Studies should include other relevant factors in their analysis of determinants of stigma, such as disease severity and skin type and/or color. In addition, studying the correlation between the level of stigmatization and the quality of life is crucial, so future studies are recommended to measure patients’ quality of life.

## Figures and Tables

**Figure 3:**
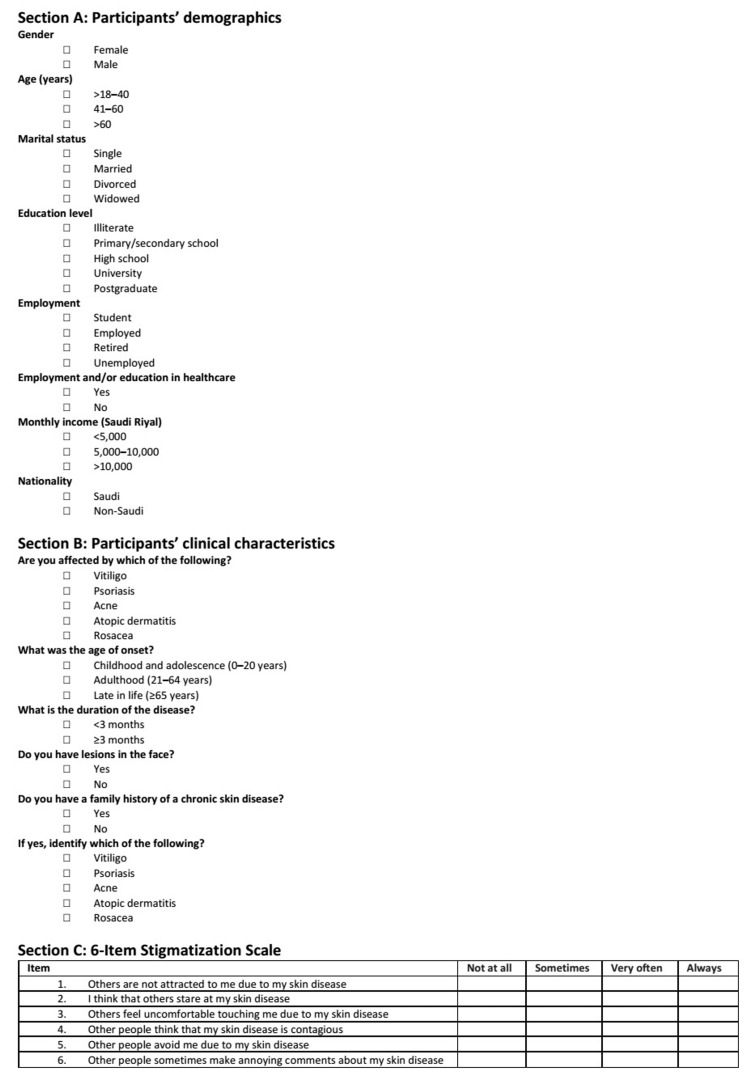
The Study Questionnaire

## Appendices

Study questionnaire
